# Plasma Procalcitonin Is Useful for Predicting the Severity of Acute Cholecystitis

**DOI:** 10.1155/2020/8329310

**Published:** 2020-01-14

**Authors:** Saben Sakalar, Engin Ozakın, Arif Alper Cevik, Nurdan Acar, Serkan Dogan, Filiz Baloglu Kaya, Taylan Kara

**Affiliations:** ^1^Eskisehir Osmangazi University Medical Center, Department of Emergency Medicine, Meselik, 26480 Eskisehir, Turkey; ^2^Departments of Internal Medicine, Emergency Medicine Section, College of Medicine and Health Sciences, United Arab Emirates University, Al Ain 17666, UAE

## Abstract

**Background:**

Acute cholecystitis is a common complication of cholelithiasis. Delayed diagnosis may constitute morbidity and mortality; therefore, early diagnosis and determining the severity of acute cholecystitis are crucial.

**Objectives:**

This study aimed to determine the validity of blood procalcitonin (PCT) levels in assessing the severity of acute cholecystitis.

**Methods:**

The Emergency Department (ED) patients diagnosed as acute cholecystitis were included in the study. Patients were allocated into three severity grades according to the Tokyo Guidelines 2013. PCT level was measured after the clinical and radiological diagnosis of acute cholecystitis in the ED.

**Results:**

Ninety-five patients diagnosed with acute cholecystitis, among them 48 of were male. Forty patients (42.1%) were allocated to grade 1, 19 (20%) to grade 2, and 36 (37.9%) to grade 3. The median values of PCT were 0.104 ng/ml, 0.353 ng/ml, and 1.466 ng/ml for grade 1, 2, and 3 patients, respectively (*p* < 0.001).

**Conclusion:**

Blood procalcitonin levels can be used to determine the severity of acute cholecystitis effectively.

## 1. Introduction

Inflammation is the primary pathological event in acute cholecystitis, which is one of the reasons for the frequent visits to the Emergency Department (ED). A bacterial infection might be added to the clinical chart as a secondary event. Obstruction caused by gallstones might develop edema, ischemia, necrosis, and ulcer in the gallbladder. The disease might confine itself in many patients. However, persistent cystic duct obstruction might lead to complications such as perforation, abscess, peritonitis, and sepsis in the patients.

Severity assessment criteria were established to reduce the mortality and the morbidity rates and to provide an early and the most convenient treatment for the patients with acute cholecystitis [1–3].

Many laboratory parameters were examined for the diagnosis and the severity assessment of acute cholecystitis. Procalcitonin (PCT), a diagnostic parameter, selectively increases in bacterial infections, sepsis, and multiple organ dysfunction syndromes. PCT does not exhibit significant elevations in viral infections, autoimmune diseases, neoplastic diseases, or operative trauma; thus, it can be used as a parameter to differentiate bacterial and nonbacterial infections, for clinical follow-up, and it can also be used as a parameter in sepsis and multiple organ dysfunction syndromes [4, 5].

In our study, we aim to detect the efficacy of PCT level in the blood in the evaluation of clinical severity in patients diagnosed with acute cholecystitis in the ED.

## 2. Materials and Methods

### 2.1. Study Design and Setting

This study was reviewed and approved by the Research Ethics Committee of the Eskisehir Osmangazi University Faculty of Medicine (reference no: 2013–15). The study lasted 15 months (June 1, 2013–September 31, 2014). The hospital is a tertiary care centre with residency training programs, and it provides 24-hour emergency service. It has 1200 beds, including 120 intensive care unit beds.

### 2.2. Study Population

The patients, who were 18 years or older and diagnosed with acute cholecystitis in accordance with the Tokyo Guidelines 2013, were included in the study. Those who are younger than 18 years, pregnant, took antibiotics within the last 72 hours, and have an additional systemic infection were excluded from the study. Written informed consent of all patients was obtained before their participation in the study.

### 2.3. Data Collection

In this study, the Tokyo Guidelines 2013 diagnostic criteria of acute cholecystitis were used to diagnose acute cholecystitis. A briefing was held to inform all emergency department physicians, who would participate in the application phase of the study, of the diagnostic criteria. In accordance with the Tokyo Guidelines 2013, in the clinical diagnosis of acute cholecystitis, the presence of one entry from each criterion was recorded as the diagnosis of cholecystitis. Patients included in the study were separated into three groups conforming to the severity grades stated in the Tokyo Guidelines 2013 (Table 1). PCT level was measured by using the B·R·A·H·M·S PCT sensitive KRYPTOR device and kit using the B·R·A·H·M·S KRYPTOR automated immunofluorescence assay with 2 ml blood collected in a biochemistry tube right after the clinical and radiological diagnosis of acute cholecystitis in the patient and before the initiation of antibiotic treatment.

Philips HD11-XE Ultrasound Machine System (convex probe: 2–5 MHz) and Toshiba Aquilion 64-Slice computed tomography (CT) scanner were used for radiological imaging. Lohexol and iopromide (90 cc/min) were used as contrast agents for CT scans. Only one radiology physician was responsible for the ultrasound evaluations and the CT scan interpretations.

We collected patients' demographic characteristics, complaints, vital signs, additional diseases, physical examination findings, laboratory results, imaging results, clinical severity grades determined by the Emergency Medicine physicians, clinical observations, and outcomes.

### 2.4. Statistical Analysis

Quantitative data, which followed a normal distribution, were expressed as means ± standard deviation, and those which did not follow a normal distribution were presented as median and percentages. Shapiro–Wilk's test was used to verify data compatibility with a normal distribution. For the comparison of the groups, which are not compatible with a normal distribution, Mann–Whitney *U* test was used when the number of groups was two and Kruskal–Wallis *H* test was used when the number of groups was three or more. Spearman correlation analysis was used to assess the direction and the strength of the relationship between variables which did not follow the normal distribution. Pearson's chi-square, Pearson's exact chi-square, and Fisher's exact chi-square tests were used to analyse the cross tables. Two-proportion tests were used to compare the proportions. IBM SPSS Statistics 21.0 and Minitab 17.0 programs were used for the application of the analyses. The *p* value of 5% or lower was set as a statistical significance criterion.

## 3. Results

Ninety-five patients diagnosed with acute cholecystitis were included in the study. Forty-eight of them (50.5%) were male. The mean age of the patients was 59.87 ± 1.96 (min: 19; max: 94). When we classified the patients for the severity grades of acute cholecystitis, 40 (42.1%) were allotted to grade 1, 19 (20%) to grade 2, and 36 (37.9%) to grade 3.

After the beginning of the symptom, the mean admission time to the ED was 37.49 ± 4.36 hour (min: 1; max: 240). We found that approximately 30% of the patients came to the ED within the first 8 hours, 19% between the 8^th^ and 12^th^ hours, 12% between the 12^th^ and 24^th^ hours, 13% between the 24^th^ and 48^th^ hours, and 15% after the 72^nd^ hour.

Murphy's sign was detected as the most common finding when the patients' physical examination findings were evaluated. Two other examination findings, which most frequently accompanied Murphy's sign, were epigastric tenderness (ET) and diffuse abdominal tenderness (DAT).

The laboratory values for PCT, white blood cell (WBC), C-reactive protein (CRP), the international normalised ratio (INR), creatinine, and platelet, which were measured to determine the severity grade of acute cholecystitis, are listed in Table 2.

Univariate analysis of demographical, physiological, and process parameters revealed values of age (*p* < 0.001), admission time (*p* < 0.001), PCT (*p* < 0.001), CRP (*p* < 0.001), INR (*p*=0.001), and platelet (*p*=0.012) as the factors which affected clinical severity (Table 3). We found a relationship between PCT values and acute cholecystitis severity levels (Table 3). We did not find a statistical difference (*p*=0.117) between the PCT values of male and female patients.

We found a difference between PCT values and age (*p* < 0.001), and cost (*p*=0.003) and admission time (*p*=0.008), but not between PCT values and the length of hospital stay (*p*=0.067).

The relationship among the patients' PCT levels, age, costs, admission time, and the length of hospital stay is demonstrated in Table 4.

## 4. Discussion

In this study, we found a relationship with PCT values and severity grades of acute cholecystitis.

Acute cholecystitis is one of the causes of abdominal pain presentations in the ED. Early diagnosis and treatment of acute cholecystitis play an important role in mortality and morbidity [3]. Acute cholecystitis might develop at any age, but its incidence is the highest at the second and the 8^th^ decade [6]. The mean age of the patients in our study was in line with the literature.

The delay in diagnosis can cause complications such as gangrene and perforation, which increase mortality and morbidity. Furthermore, the method of treatment and surgery choice might change in relation to the admission time to the ED after the beginning of the symptoms [7]. We observed a higher grade of severity in our patients when their admission time was prolonged. However, the admission time did not affect the method of surgery.

In the literature, the specificity of Murphy's sign was stated as 35–98% and its sensitivity as 63–96% for acute cholecystitis in the physical examination [8, 9]. Murphy's sign identified in 85 (89.5%) patients in our study also appeared as the most frequently seen physical examination finding.

In acute cholecystitis, WBC count increases, but it is not specific to this condition and leukocytosis is not reliable for diagnosis especially in patients admitted to the ED [10]. The median WBC value of our patients was 11000/mm³. When we examine the relationship between the clinical grade and WBC count, the mean was measured 10550/mm³ in grade 1, 12900/mm³ in grade 2, and 11280/mm³ in grade 3. In addition to severe acute cholecystitis [11] and gangrenous cholecystitis [12], leukocyte counts were also found higher in the elderly than young patients [13]. Unlike stated in the literature, the mean WBC count of the patients included in our study was found lower. Furthermore, we did not see a statistical difference between the WBC count and severity grade. The patient group included in the study and their number might be the reason for this result. We also did not have patients with severe complications.

Ultrasound findings, which indicate acute cholecystitis, alongside the elevation in CRP levels (3 mg/dl or higher) have a 97% sensitivity, 76% specificity, and 95% positive-predictive value in the diagnosis of acute cholecystitis [1]. CRP level was measured lower than the mentioned value in grade 1 and grade 2 patients; however, its median value was measured 9.07 mg/dl in grade 3 patients. In our study, although the CPR levels supported the diagnosis insufficiently, higher levels might be used as a parameter to assess the severity levels.

Although some studies defend the uselessness of high PCT without having infectious complications [14], PCT was found useful in demonstrating the severity of inflammation in acute pancreatitis in a study examining disease severity and PCT level, which was used to evaluate the inflammatory response [15, 16]. A study, which analysed the relationship of the severity of acute appendicitis with PCT and CRP, detected an elevation in both parameters in complicated acute appendicitis cases [17]. A study investigating DM patients showed that PCT is better than CRP and WBC to anticipating sepsis. Some studies investigated PCT and cholangitis severity [18–20]. However, the literature is scarce for PCT and cholecystitis [21]. Yuzbasioglu et al. showed PCT could be used as a laboratory parameter to define acute cholecystitis severity [21]. They also described a positive relationship between PCT and CRP, leukocyte, and sedimentation levels. In our research, PCT level was within the normal range of values in 52 patients (53.9%) and within a range of values between severe sepsis and septic shock in 17 patients (18.7%). The data obtained from our patients demonstrated that PCT levels could not be used as a diagnostic parameter in acute cholecystitis patients at the ED, yet it could be used as an efficient parameter in the severity assessment. Our study also revealed that the severity level was directly proportional to INR elevation, but it was inversely proportional to TR count, and their relationship was statistically significant. We also did not encounter a study which shows the relation of disease severity with INR and platelets count in the literature.

In addition to the easing decision-making process, TG07, TG13, and TG18 also provide basic criteria, helping to grade acute cholecystitis severity [1–3]. The patients with severe acute cholecystitis varied from 1.2% to 6% in studies using TG07 [22, 23]. Yokoe et al. reported that the severe acute cholecystitis patients' rate was 17.2%, according to TG13.9 [24]. In this study, 37.9% of our patients were in grade 3. We think that this result is because of the referral of patients who have comorbidities, severe clinical symptoms to our tertiary care institution.

There are various treatment options such as mild cholecystitis patients can be a candidate for laparoscopic cholecystectomy, moderate cholecystitis patients can be treated with laparoscopically or percutaneous cholecystostomy, and severe cases require percutaneous cholecystostomy [2]. According to the Tokyo Guideline 2018 (TG18), laparoscopic cholecystectomy is recommended for mild and moderate acute cholecystitis patients [25]. However, it is reported that laparoscopic cholecystectomy can be difficult in severe patients because of edema and adhesions [25]. Therefore, it may be foreseen that PCT levels can be valuable in helping the decision of the surgical approach. In our study, 47 of the hospitalised patients (55.9%) underwent surgical treatment and laparoscopy was performed on all patients. Medical treatment was preferred for the remaining 37 patients. The rejection of emergency surgery by the patient or his/her relatives, their request for only medical treatment, and high-risk factors due to the additional diseases affected the preference of treatment. We did not perform laparotomy on any of the patients; thus, the relationship between PCT and treatment methods could not be analysed.

In another study, the incidence of gallbladder perforation in acute cholecystitis has been reported to range from 2 to 15%. The study also stated that it could not clinically differentiate from acute cholecystitis easily, its diagnosis was difficult before surgery, and the late surgical interventions due to the lack of early diagnosis in these cases were related to the increased morbidity, mortality (70%), longer intensive care time, and hospital stay [26]. In our study, according to the results of surgical and pathological reports, no patients developed perforation. Thus, the relationship between PCT and the complication could not be evaluated.

Today, the mortality rates were lower in gallstone disease as compared to the previous years. In our study, only one diagnosed patient died. This patient did not undergo surgery due to the additional conditions and also did not show a response to the medical treatment. The PCT level in this patient was measured as 85.95 ng/ml.

PCT level also affects the cost of the patient. Elevation of PCT level results in a prolonged hospital stay and consequently the cost increases.

### 5.1. Limitations

All ultrasound imagings were not performed by the same radiology assistant was regarded as a limitation of this study.

The patients were not hospitalised according to the hospitalisation criteria; thus, they were admitted to the empty and available spaces. This situation might be seen as a limitation of our study.

The patients were included in the study after they were diagnosed with acute cholecystitis; thus, the consequent lack of a control group in our study hindered us to state the threshold value particularly in laboratory parameters. This might be regarded as a limitation of our study.

## 6. Conclusion

Acute cholecystitis, mistaken for many diseases, is the inflammation of the gallbladder and one of the most common reasons for acute abdominal pain. The higher the severity level of the patients, the higher the PCT levels in patients. We found a difference between the patients' PCT values and their severity grades of acute cholecystitis. PCT can be used as an effective laboratory method in the severity assessment of acute cholecystitis, yet we need additional studies, which would include a higher number of the patient population.

## Figures and Tables

**Table 1:** Criteria for evaluation of acute cholecystitis severity^*∗*^.

Grade 3	Grade 2	Grade 1
Cardiovascular dysfunction: hypotension requiring treatment with dopamine ≥5 *μ*g/kg per min, or any dose of norepinephrine	Elevated WBC count (>18,000/mm^3^)	Does not meet criteria of grade 3 and grade 2 acute cholecystitis
Neurological dysfunction: decreased level of consciousness	Palpable tender mass in the right upper abdominal quadrant	Healthy patients with no organ dysfunction and mild inflammatory changes
Respiratory dysfunction: PaO_2_/FiO_2_ ratio <300	Duration of complaints >72 h^a^	
Renal dysfunction: oliguria, creatinine >2.0 mg/dl	Marked local inflammation (gangrenous cholecystitis, pericholecystic abscess, hepatic abscess, biliary peritonitis, emphysematous cholecystitis)	
Hepatic dysfunction: PT‐INR > 1.5		
Hematological dysfunction: platelet count <100,000/mm^3^		

^*∗*^According to the Tokyo Guidelines updated in 2013.

**Table 2:** Laboratory median values of patients.

Laboratory value	Median	Q1–Q3
PCT (ng/ml)	0.333	0.078–1.67
WBC (×10^9^/L)	11.0	7.7–14.3
CRP (mg/L)	2.39	0.84–8.91
INR	1.07	1.02–1.14
Creatinine (mg/dl)	0.87	0.68–1.1
Platelets (×10^9^/L)	223	169–272

**Table 3:** Univariate analysis of factors affecting the severity of acute cholecystitis and relationship between procalcitonin values and acute cholecystitis severity levels.

	Grade 1 median (Q1–Q3)	Grade 2 median (Q1–Q3)	Grade 3 median (Q1–Q3)	*p*
Gender				0.436
Male	17 (42.5%)	11 (57.9%)	20 (55.6%)	
Female	23 (57.5%)	8 (42.1%)	16 (44.4%)	
Age	49.50 (36–61.75)	72.00 (48–79)	72.50 (62.75–81)	**<0.001**
Emergency department admission time (hour)	9.50 (4–15)	72 (17–96)	36 (11–72)	**<0.001**
PCT	0.104 (0.03–0.65)	0.353 (0.09–1.61)	1.466 (0.17–9.00)	**<0.001**
WBC	10550 (8342–13100)	12900 (8010–18720)	11280 (6777–18225)	0.338
CRP	1.06 (0.31–2.43)	1.87 (0.97–4.29)	9.07 (2.90–17.82)	**<0.001**
INR	1.04 (1.00–1.09)	1.09 (1.04–1.16)	1.11 (1.05–1.21)	**0.001**
Creatinine	0.82 (0.65–0.99)	1.00 (0.74–1.36)	0.94 (0.66–1.65)	0.099
Platelet	251 (209–284)	218 (163–272)	193 (97–260)	0.012
Hospital stay (day)	7 (4.25–9.75)	7 (4–8)	8.5 (6–11)	0.109
Cost (Euro)	183 (88–282)	246 (110–366)	257 (183–431)	0.027

**Table 4:** Relationship between PCT, age, cost, admission time, and length of hospital stay.

	PCT	Age	Cost	Admission time	Length of hospital stay
PCT	—	0.481 <0.001	0.299 0.003	0.270 0.008	0.189 0.067
Age		—	0.413 <0.001	0.349 0.001	0.219 0.033
Cost			—	0.122 0.238	0.717 <0.001
Length of hospital stay				—	0.014 0.889

Spearman correlation test.

## Data Availability

The data used to support the findings of this study are available from the corresponding author upon request.
